# The impact of intensive management on pain intensity in patients with rheumatoid arthritis and psoriatic arthritis: secondary analysis of three clinical trials

**DOI:** 10.1186/s41927-025-00493-z

**Published:** 2025-05-21

**Authors:** Fowzia Ibrahim, David L. Scott, Ian C. Scott

**Affiliations:** 1 Centre for Rheumatic Diseases, Department of Inflammation Biology, School of Immunology and Microbial Sciences, Faculty of Life Sciences and Medicine, King’s College London Cutcombe Road, London, SE5 9RJ UK; 2 Department of Rheumatology, Kings College Hospital, Denmark Hill, London, SE5 9RS UK; 3 Primary Care Centre Versus Arthritis, School of Medicine, Keele University, Keele, UK; 4 Haywood Academic Rheumatology Centre, Haywood Hospital, Midlands Partnership University NHS Foundation Trust, High Lane, Burslem, Staffordshire, UK

**Keywords:** Rheumatoid arthritis, Psoriatic arthritis, Clinical trial, Pain, Disease-modifying anti-rheumatic drug

## Abstract

**Background:**

Understanding the impact of intensive treatment on pain in patients with rheumatoid arthritis (RA) and psoriatic arthritis (PsA) is crucial to informing the application of evidence-based arthritis pain care. The impact of intensive treatment on inflammatory arthritis pain has received relatively limited attention. We addressed this through a detailed secondary analysis of three trials evaluating varying intensities of disease-modifying anti-rheumatic drug treatment. We considered a range of pain outcomes of clinical relevance to patients, including the achievement of mild endpoint pain scores and clinically-meaningful pain reductions.

**Methods:**

The trials comprised MIPA in PsA, CARDERA in early RA, and TITRATE in established RA. Pain was measured using a 100-mm pain intensity visual analogue scale (VAS). The impact of intensive treatment on (a) patients achieving “mild” endpoint pain intensity scores (of ≤ 34), b) mean changes in pain intensity scores, and (c) patients achieving ≥ 30% reductions in pain intensity scores was evaluated using t-tests, chi-squared tests, and regression models (with the latter adjusting for relevant potential confounding variables).

**Results:**

From MIPA, CARDERA, and TITRATE 128, 379, and 258 patients had endpoint outcome data available and were included in this secondary analysis. In all trials, significantly more patients achieved mild endpoint pain intensity scores with intensive vs. control treatment (MIPA 70% vs. 42%, *P* = 0.003; CARDERA 71% vs. 56%, *P* = 0.011; TITRATE 67% vs. 50%, *P* = 0.008). In the two trials employing the most intensive management strategies (CARDERA; TITRATE) overall reductions in pain scores were significantly greater (6.6 to 6.8 units in adjusted linear regression models), and significantly more achieved ≥ 30% reductions in pain with intensive vs. control treatment (adjusted logistic regression models: CARDERA odds ratio [OR] 1.9, *P* = 0.009; TITRATE OR 2.2, *P* = 0.002).

**Conclusions:**

Intensive treatment is an important component of improving pain in patients with active RA and PsA. Our findings support EULAR guidance that optimising disease activity is crucial for pain control. As approximately one third of patients receiving active treatment had moderate/high endpoint pain intensity levels across trials, additional pain management strategies that complement intensive treatment are needed.

**Trial registration:**

Current Controlled Trials CARDERA ISRCTN32484878 (25/10/2000), MIPA ISRCTN54376151 (04/02/2002), and TITRATE ISRCTN70160382 (16/1/2014).

## Background

Chronic pain is a major challenge for patients with rheumatoid arthritis (RA) and psoriatic arthritis (PsA). Patients with RA often have daily pain [1] and rate pain as their preferred area for health improvement [2]. Patients with PsA report joint and spine pain as their most bothersome symptoms [3]. Improving pain management in patients with RA and PsA is, therefore, a research priority, as detailed in the Versus Arthritis Pain Roadmap, whose central vision is “an end to pain” in patients with arthritis and other musculoskeletal conditions [4].

Real-world studies show pain management in RA and PsA focuses on substantial and sustained opioid prescribing. In England, a regional electronic health record study reported that 40–50% of patients with PsA and RA received an opioid prescription in 2015 [5]. Other countries have comparable levels of opioid prescribing [6, 7]. Yet there is no trial evidence that opioids are effective in RA or PsA beyond 6-weeks (with little effect observed in historical short-term trials in RA) [8], and they have many harms like serious infections [9] and fractures [10].

Intensive management – using combination synthetic, biologic, or targetted synthetic disease-modifying anti-rheumatic drugs (DMARDs) or undertaking regular assessments with the application of treat-to-target principles – reduces disease activity [11], improves function [12], and reduces radiological damage [13] in inflammatory arthrits. However, its impact on pain has received relatively little attention. Highly cited trials of DMARDs in inflammatory arthritis either overlook pain as a seperate outcome [14, 15] or only report intensive treatment effects on mean changes in pain scores [16]. The often bimodal distribution of pain intensity scores – with many patients with chronic pain having either very good or very little pain relief in response to treatment – means considering average differences in pain scores post-interventions may underestimate their effectiveness, obscuring whether a substantial minority achieve good pain relief [17]. Conventional analytical approaches also overlook patients’ concerns about chronic pain care, who want substantial reductions in pain levels and the achievement of “no worse than mild” pain [18].

Understanding the impact of intensive management on pain in patients with inflammatory arthritis is important. If it improves patients’ pain from perspectives which matter to them – achieving mild pain levels and substantial pain reductions – this would support moving the focus of pain care from prescribing opioids towards conventional DMARDs and other treatments of proven efficacy. We addressed this evidence gap in secondary analyses of three randomised controlled trials (RCTs) evaluating the impact of varying levels of intensive management on pain intensity outcomes from a range of different perspectives in patients with RA and PsA.

## Methods

### Included trials

We included RCTs meeting the following criteria: (a) designed and completed after 2000 (ensuring contemporary relevance), (b) conducted by an author of the current secondary analysis, (c) enrolling patients with active RA or PsA, (d) collecting data on pain intensity levels, and (e) using approved DMARDs in their licensed indications. Three RCTs met these criteria: Methotrexate in Psoriatic Arthritis (MIPA) [19], Combination Anti-Rheumatic Drugs in Early Rheumatoid Arthritis (CARDERA) [20], and Treatment Intensities and Targets in Rheumatoid Arthritis Therapy (TITRATE) [21]. The primary pre-specified analyses from each trial have previously been published, and the findings from this secondary analysis were presented at the British Society for Rheumatology annual conference [22].

### Trial treatments, durations, and assessments

MIPA randomised patients with active PsA to methotrexate monotherapy or placebo. Patients were assessed monthly and methotrexate up-titrated according to disease activity levels to 15-25 mg/week. Its primary outcome was PsA response criteria at 6 months. CARDERA randomised patients with early active RA to methotrexate monotherapy, methotrexate and ciclosporin, methotrexate and intensive prednisolone for 9 months, or all three treatments. Its primary outcome was new erosions at 24 months. For this secondary analysis, we classified triple therapy as active treatment and other treatments as controls. TITRATE randomised patients with moderately active RA to intensive treatment (monthly appointments with specially trained healthcare professionals incorporating clinical assessments and protocolised medication up-titration targeting remission) or standard care (optimising DMARDs without specific treatment/follow-up plans). Its primary outcome was disease activity score for 28 joints using the erythrocyte sedimentation rate (DAS28-ESR) remission at 12 months. All trials recorded patients’ age, sex, and disease duration at baseline, and DAS28-ESR and pain intensity scores – assessed using a 100 mm visual analogue scale (VAS) – at baseline and endpoints.

### Pain intensity outcomes

We considered the impact of intensive management on pain intensity using three complementary pain outcomes: (1) the proportion of patients at trial endpoints with “mild” pain intensity based on a VAS cut-off of ≤ 34 (shown in chronic musculoskeletal pain to best identify patients describing their pain as mild [23]); (2) mean changes in pain intensity scores between baseline and endpoints; and (3) the proportion of patients achieving reductions in pain intensity scores between baseline and endpoints of ≥ 30%. This latter cut-off is recommended by the Initiative on Methods, Measurement, and Pain Assessment in Clinical Trials of chronic pain [24], and is based on an analysis of the relationship between changes in pain intensity and patient reports of overall improvement in ten clinical trials of chronic pain, which demonstrated that a reduction of 30% was able to accurately discriminate between people rating their condition as “much or very much improved” or not [25]. An additional analysis reported the proportion of patients receiving active vs. control treatments with initial moderate-to-high pain intensity scores (> 34) who progressed to have mild pain intensity scores (≤ 34) at trial endpoints and vice versa.

### Statistical analyses

We analysed patients with complete trial end-point data. Each trial was analysed separately using IBM SPSS version 27. Baseline patient characteristics and outcomes were summarised using means (with standard deviations [SD]) and numbers (with proportions) as appropriate. Mean (SD) endpoint pain intensity scores in each trial were also reported.

Differences between treatment groups at baseline and endpoint were assessed using unpaired Student’s t-tests (means) and Chi-squared tests (categorical data). Linear regression models further tested the association between trial treatment as the explanatory variable and change in pain intensity scores as the response variable. Logistic regression models further tested the association between trial treatment as the explanatory variable and achieving endpoint pain scores ≤ 34 or a change in pain scores ≥ 30% as the response variable. Multivariable regression models included the variables age, sex, and baseline pain intensity scores. An additional analysis calculated Pearson’s correlations between changes in DAS28 and pain intensity scores from baseline to endpoint in each trial, to explore the extent to which improving disease activity may improve pain.

### Ethical approvals

The trials were approved by the following Research Ethics Committees: MIPA and CARDERA - South-East Multi-Centre Research Ethics Committee; TITRATE - West London & GTAC National Research Ethics Service Committee. All patients gave written informed consent.

## Results

### Patient numbers, characteristics, baseline assessments, and end point pain intensity scores

MIPA, CARDERA, and TITRATE randomised 221, 467, and 335 patients to treatment; 128 (58%), 379 (81%), and 258 (77%) had endpoint outcome data available and were included in this secondary analysis. Table 1 shows their baseline characteristics and outcome measures.

**Table 1:** Baseline patient characteristics and outcome measures

	MIPA		CARDERA		TITRATE	
	*Control*	*Active*	*Control*	*Active*	*Control*	*Active*
Number of Patients	59	67	282	97	124	134
Female	21 (36%)	29 (43%)	196 (70%)	63 (65%)	99 (80%)	114 (85%)
Mean Age in Years	51 (10)	47 (11)	54 (12)	55 (12)	57 (12)	57 (12)
Mean Disease Duration in Years	6 (9)	5 (6)	0.4 (0.5)	0.4 (0.5)	5 (5)	6 (7)
Mean DAS28-ESR	5.0 (1.4)	5.1 (1.5)	5.8 (1.3)	5.6 (1.3)	4.3 (0.5)	4.3 (0.5)
Mean HAQ	1.1 (0.7)	1.0 (0.7)	1.6 (0.7)	1.5 (0.7)	1.2 (0.7)	1.2 (0.7)
Mean Pain Intensity	47 (25)	38 (22)	48 (25)	44 (25)	43 (23)	41 (22)
Low Pain Intensity Scores (≤ 34)	25 (42%)	32 (48%)	95 (34%)	38 (39%)	43 (35%)	53 (40%)

Data are mean (SD) or number (%). Baseline pain intensity scores missing in 2 patients in MIPA. DAS28-ESR = disease activity score for 28 joints using the erythrocyte sedimentation rate (DAS28-ESR). HAQ = health assessment questionnaire. Pain intensity scores measured using 100 mm visual analogue scale

Baseline mean pain intensity scores were numerically lower and the proportion of patients with mild pain intensity scores numerically higher in the active treatment arms of all trials. However, there were no significant differences in baseline characteristics or outcome measures between patients receiving control or active treatments in any trial.

In all trials mean endpoint pain intensity scores were lower in patients receiving active compared to control treatment (MIPA 28 (SD 26) vs. 37 (SD 22); CARDERA 26 (SD 23) vs. 35 (SD 28); TITRATE 27 (SD 25) vs. 38 (SD 29)).

### Mild End-Point pain intensity scores

In all trials significantly greater proportions of patients achieved mild endpoint pain intensity scores of ≤ 34 units with active than control treatments (Table 2): in MIPA 70% vs. 42% (*P* = 0.003); in CARDERA 71% vs. 56% (*P* = 0.011); and in TITRATE 67% vs. 50% (*P* = 0.008). Univariate logistic regression models also showed significant relationships between treatment and mild endpoint pain scores (Table 3). Multivariable models showed the associations remained significant after treatment effects were adjusted for age, sex, and baseline pain: odds ratios were 2.8 (*P* = 0.012), 1.9 (*P* = 0.019) and 2.1 (*P* = 0.006) in MIPA, CARDERA, and TITRATE.

**Table 2:** Association between intensive management and endpoint assessments of pain intensity in each trial

		MIPA			CARDERA			TITRATE		
		*Control*	*Active*	*Statistical Significance*	*Control*	*Active*	*Statistical Significance*	*Control*	*Active*	*Statistical Significance*
Low Pain Intensity Score (≤ 34)		25 (42%)	47 (70%)	χ^2^ = 9.9; *P* = 0.003	157 (56%)	69 (71%)	χ^2^ = 6.5; *P* = 0.011	62 (50%)	90 (67%)	χ^2^ = 7.1; *P* = 0.008
Mean Change in Pain Intensity Score		10 (27)	11 (27)	NS	13 (28)	18 (28)	NS	5 (30)	13 (30)	*P* = 0.036
Reductions In Pain Intensity Score ≥ 30%		26 (45%)	37 (55%)	NS	138 (49%)	61 (63%)	χ^2^ = 5.1; *P* = 0.024	49 (40%)	78 (58%)	χ^2^ = 8.3; *P* = 0.004

Data are mean (SD) or number (%). DF = 1 in all χ^2^ analyses; means compared between active and control treatment using t-tests; number achieving low or substantial reductions in pain intensity scores between active and control treatment compared using chi-squared tests; NS = non-significant (*P* > 0.05)

**Table 3:** Regression analysis of impact of intensive treatment on endpoint pain and changes in pain

Outcome	Trial	Unadjusted		Adjusted	
		*Difference*	*Significance*	*Difference*	*Significance*
***Linear Regression Models***					
Change In Pain	*TITRATE*	7.8 (0.5, 15.1)	*P* = 0.036	9.4 (3.0, 15.8)	*P* = 0.004
	*CARDERA*	4.8 (-1.7, 11.3)	*P* = 0.147	6.8 (1.1, 12.4)	*P* = 0.019
	*MIPA*	1.6 (-8.0, 11.3)	*P* = 0.742	6.6 (-1.8, 15.1)	*P* = 0.124
***Logistic Regression Models***					
Endpoint Pain ≤ 34	*TITRATE*	2.0 (1.2, 3.4)	*P* = 0.005	2.1 (1.2, 3.6)	*P* = 0.006
	*CARDERA*	2.0 (1.2, 3.2)	*P* = 0.008	1.9 (1.1, 3.2)	*P* = 0.019
	*MIPA*	3.2 (1.5, 6.7)	*P* = 0.002	2.8 (1.2, 6.1)	*P* = 0.012
Reductions In Pain ≥ 30	*TITRATE*	2.1 (1.3, 3.5)	*P* = 0.003	2.2 (1.3, 3.7)	*P* = 0.002
	*CARDERA*	1.8 (1.1, 2.8)	*P* = 0.018	1.9 (1.2, 3.1)	*P* = 0.009
	*MIPA*	1.5 (0.7, 3.1)	*P* = 0.247	1.7 (0.8, 3.6)	*P* = 0.158

95% confidence intervals shown. Adjusted models include the covariates age, sex, and baseline pain intensity VAS

### Mean changes in pain intensity scores

The trials differed in the impact of intensive treatment on mean changes in pain intensity scores (Table 2). In MIPA mean changes in pain intensity scores over the trial period were similar with active (-11) and control (-10) treatment. In CARDERA mean changes in pain intensity scores were greater with active (-18) than control (-13) treatment, though this difference was not statistically significant. In TITRATE mean changes in pain intensity scores were significantly higher with active (-13) than control (-5) treatments (t-test *P* = 0.036).

Univariate and multivariable linear regression models confirmed the findings in MIPA and TITRATE (Table 3). In MIPA unadjusted and adjusted analyses showed no significant differences between treatment groups. In TITRATE unadjusted and adjusted models both showed significant differences between treatment groups (*P* = 0.036 and 0.004). In CARDERA the unadjusted model showed no difference between treatment groups but there was a significant treatment effect in the adjusted model (*P* = 0.019).

### Reductions in pain intensity scores of ≥ 30%

The trials differed in the impact of treatment on the proportion of patients having clinically meaningful reductions in pain intensity scores (Table 2). In MIPA proportionally more patients (10%) had ≥ 30% reductions in pain intensity scores with active treatment, but this difference was not statistically significant. Both CARDERA and TITRATE showed significant differences with treatment in the numbers of patients achieving large reductions in pain intensity scores. In CARDERA 63% of patients had a ≥ 30% reduction with active treatment compared with 49% of controls (*P* = 0.024). In TITRATE 58% of patients had a ≥ 30% reduction with active treatment compared with 40% of controls (*P* = 0.004).

Univariate and multivariable logistic regression models confirmed these associations (Table 3). There were no significant differences between groups in MIPA in either model. There were significant differences in CARDERA (unadjusted model *P* = 0.018; adjusted model *P* = 0.009) and in TITRATE (unadjusted model *P* = 0.003; adjusted model *P* = 0.002).

### Changes in pain intensity status from baseline to endpoint

In all trials, a greater proportion of people with high pain intensity scores at baseline subsequently had low pain intensity scores at endpoints with intensive vs. control treatment (Table 4; MIPA 60% vs. 32%; CARDERA 61% vs. 43%; TITRATE 59% vs. 38%). Conversely, a greater proportion of people with low pain intensity scores at baseline subsequently had high pain intensity scores at endpoints with control vs. intensive treatment (MIPA 44% vs. 19%; CARDERA 20% vs. 13%; TITRATE 28% vs. 21%).

**Table 4:** Proportion of patients with moderate/high pain at baseline achieving mild pain at endpoint and vice versa

Trial	Group	Initial Pain	Final Pain	
			Moderate/High > 34	Mild ≤ 34
MIPA	*Control*	Moderate/High > 34 (*n* = 34)	*n* = 23 (68%)	*n* = 11 (32%)
		Mild ≤ 34 (*n* = 25)	*n* = 11 (44%)	*n* = 14 (56%)
	*Intensive*	Moderate/High > 34 (*n* = 35)	*n* = 14 (40%)	*n* = 21 (60%)
		Mild ≤ 34 (*n* = 32)	*n* = 6 (19%)	*n* = 26 (81%)
CARDERA	*Control*	Moderate/High > 34 (*n* = 187)	*n* = 106 (57%)	*n* = 81 (43%)
		Mild ≤ 34 (*n* = 95)	*n* = 19 (20%)	*n* = 76 (80%)
	*Intensive*	Moderate/High > 34 (*n* = 59)	*n* = 23 (39%)	*n* = 36 (61%)
		Mild ≤ 34 (*n* = 38)	*n* = 5 (13%)	*n* = 33 (87%)
TITRATE	*Control*	Moderate/High > 34 (*n* = 81)	*n* = 50 (62%)	*n* = 31 (38%)
		Mild ≤ 34 (*n* = 43)	*n* = 12 (28%)	*n* = 31 (72%)
	*Intensive*	Moderate/High > 34 (*n* = 81)	*n* = 33 (41%)	*n* = 48 (59%)
		Mild ≤ 34 (*n* = 53)	*n* = 11 (21%)	*n* = 42 (79%)

### Correlations between changes in pain intensity and disease activity scores

There were moderate correlations between changes in pain intensity and DAS28 scores in all trials (MIPA: *r* = 0.46; CARDERA: *r* = 0.57; TITRATE: *r* = 0.59).

## Discussion

Our secondary analysis of three RCTs in patients with active RA or PsA shows that all evaluated forms of intensive management had some beneficial effect on pain intensity scores. More patients achieved mild endpoint pain intensity scores with active treatment in all trials. In the two trials employing the most intensive management strategies (combination synthetic DMARDs with high-dose prednisolone or monthly assessments with synthetic/biologic DMARD up-titration and psychological support) significantly greater reductions in pain intensity levels were also seen with active treatment when evaluated using regression models with overall reductions in pain scores being higher, and a greater proportion achieving clinically meaningful improvements in pain. Taken together, our findings support the use of intensive management to improve pain in patients with active RA or PsA, and the EULAR arthritis pain guideline recommendation that treating disease activity is “crucial” to achieving pain control [26], with the caveat that as changes in disease activity have only moderate correlations with changes in pain over time, additional biopsychosocial treatment approaches are required.

Whilst the primary publications for TITRATE [21] and MIPA [19] provided details around the impacts of active vs. control treatment on mean pain intensity scores over the trial period, these were not reported in the primary publication for CARDERA [20], and our secondary analysis employed a range of pain outcomes to better understand the impact of treatment from the perspective of patients having “no worse than mild” pain alongside clinically important reductions in pain levels (of ≥ 30%), which are advocated for use in chronic pain trials. It is not unexpected that greater improvements were not seen with active vs. control treatment for all the pain outcomes we evaluated, as they represent distinct but complementary outcomes. The fact that mean changes in pain intensity scores as an outcome in chronic pain trials has potential limitations is well described, with changes in pain scores in response to analgesic treatments often being bimodal, meaning that small mean differences in analgesia between treatment arms can obscure the fact that a significant minority achieve very good pain improvements [17]. We consider that our finding of intensive management leading to over two-thirds of patients achieving mild endpoint pain intensity scores (compared to under half receiving control treatment) is of most clinical importance, with Moore, Straube, and Adlington arguing – based on studies evaluating pain thresholds against global questions of response – that the achievement of “no worse than mild pain” is the key outcome of interest in chronic pain trials [18]. Additionally, in CARDERA and TACIT intensive management led to 58–63% of patients reducing their pain intensity scores by ≥ 30% (compared to 40–49% with control treatment); reductions in pain scores were 7–10 units more with active treatment after accounting for relevant confounding variables. We consider that broadening the pain outcomes assessed provides crucial complementary information, which is easily understood by both clinicians and patients.

It is challenging to compare the efficacy of intensive management on pain intensity to that of opioids due to the heterogeneity of pain outcomes used in trials in this area. A Cochrane review highlighted the paucity of data around opioid efficacy in RA [8]. It identified 11 trials: they had high risks of bias, were of short duration, often assessed unconventional opioids, and were conducted pre-2006. The authors concluded that “at best” weak evidence existed in favour of short-term opioid efficacy for pain in RA.

One concern from our own findings is that despite using intensive DMARD management approximately one third of patients receiving active treatment had moderate or high pain intensity levels at trial endpoints. Furthermore, in a minority of patients receiving intensive treatment, pain levels can worsen (with 13–21% of patients receiving active treatment in CARDERA/MIPA/TITRATE moving from mild pain at baseline, to moderate/high pain at endpoints). This highlights the need to use multiple strategies to improve pain. EULAR guidelines for arthritis pain care advocate a stepped biopsychosocial approach, which both focusses on optimising disease activity and also addresses mood, sleep, weight, and biomechanical factors [26]. TITRATE was the only trial that included non-drug care, providing nurse-delivered “treatment support” focussing on pain and fatigue management, physical activity, medication adherence, sleep, and mood. Whilst it is impossible to discern the effects of these intervention components from its other approaches (monthly assessments and DMARD escalation targeting remission) the largest effect sizes for pain intensity were seen in TITRATE. Interestingly, recent studies with baricitinib, a targeted synthetic DMARD, have highlighted its significant impact on reducing pain intensity [27], suggesting growing awareness of the impact of these drugs on pain.

It is noteworthy that the least benefits of intensive treatment on pain were seen in the MIPA trial. This trial has several key differences compared with CARDERA and TACIT. The two most relevant are that the management approach used (monthly assessments with methotrexate up-titrated according to disease activity levels) was less intensive, and did not result in reductions in synovitis compared with control treatment (although it did lead to reductions in patient and assessor global assessments). It is likely that these two factors explain the less clear-cut impacts of intensive treatment on pain observed in MIPA.

Our study’s strengths are its inclusion of large numbers of patients from multiple specialist centres across England using identical clinical assessments (ensuring generalisability), and replication of beneficial effects of intensive management on pain intensity across trials. Its limitations are its post-hoc design, inclusion of patients with complete endpoint data (including all patients using multiple imputation methods may give different perspectives, although from the viewpoint of clinical relevance patients with endpoint data are most important), consideration of only one pain dimension (intensity), and inclusion of less commonly used treatment regimens (ciclosporin and high-dose prednisolone) in CARDERA.

## Conclusions

We conclude that, across a range of trial designs and settings, there is good evidence that intensive management using DMARDs improves pain intensity in patients with active RA and PsA over 6 months or longer in a manner that is clinically relevant to them. This contrasts with the limited evidence for opioid efficacy in short-term historical trials. Our analysis supports EULAR guidance that optimising disease activity is crucial for pain control.

## Data Availability

The data generated during these trials are not publicly available because consent to make their data publicly available was not specifically sought from trial participants. Anonymised summary data will be available from the authors for inclusion in meta-analyses and other relevant similar academic endeavours.

## Abbreviations

CARDERA: Combination Anti-Rheumatic Drugs In Rheumatoid Arthritis Trial
DAS28-ESR: Disease Activity Score For 28 Joints With Erythrocyte Sedimentation Rate
DMARDs: Disease-Modifying Anti-Rheumatic Drugs
EULAR: European Alliance of Associations for Rheumatology
HAQ: Health Assessment Questionnaire
MIPA: Methotrexate In Psoriatic Arthritis Trial
PsA: Psoriatic Arthritis
RA: Rheumatoid Arthritis
RCT: Randomised Controlled Trial
SD: Standard Deviation
TITRATE: Treatment Intensities and Targets In Rheumatoid Arthritis Therapy Trial
VAS: Visual Analogue Scale
